# Influence of GeO_2_ Content on the Spectral and Radiation-Resistant Properties of Yb/Al/Ge Co-Doped Silica Fiber Core Glasses

**DOI:** 10.3390/ma15062235

**Published:** 2022-03-17

**Authors:** Yiming Zhu, Yan Jiao, Yue Cheng, Chongyun Shao, Chunlei Yu, Ye Dai, Lili Hu

**Affiliations:** 1 Department of Physics, Shanghai University, Shanghai 200444, China; zym013579@gmail.com (Y.Z.); chengyue@siom.ac.cn (Y.C.); 2 Key Laboratory of High Power Laser Materials, Shanghai Institute of Optics and Fine Mechanics, Chinese Academy of Sciences, Shanghai 201800, China; jiaoyan@siom.ac.cn (Y.J.); hulili@siom.ac.cn (L.H.); 3 Hangzhou Institute for Advanced Study, University of Chinese Academy of Sciences, Hangzhou 310024, China

**Keywords:** Yb-doped silica glass, radiation resistance, anti-darkening, color center

## Abstract

In this study, Yb/Al/Ge co-doped silica fiber core glasses with different GeO_2_ contents (0–6.03 mol%) were prepared using the sol–gel method combined with high-temperature sintering. The absorption, fluorescence, radiation-induced absorption, continuous-wave electron paramagnetic resonance spectra, and fluorescence decay curves were recorded and analyzed systematically before and after X-ray irradiation. The effects of GeO_2_ content on the valence variations of Yb^3+^/Yb^2+^ ions, spectral properties of Yb^3+^ ions, and radiation resistance of Yb/Al/Ge co-doped silica glasses were systematically studied. The results show that even if the GeO_2_ content of the sample is relatively low (0.62 mol%), it can inhibit the generation of Yb^2+^ ions with slight improvement in the spectral properties of Yb^3+^ ions in the pristine samples and effectively improve its radiation resistance. Direct evidence confirms that the generation of trapped-electron centers (Yb^2+^/Si-E’/Al-E’) and trapped-hole centers (Al-OHC) was effectively inhibited by Ge co-doping. This study provides a theoretical reference for the development of high-performance, radiation-r esistant Yb-doped silica fibers.

## 1. Introduction

Over the past few decades, rare-earth (RE)-doped silica fiber lasers and amplifiers have been studied extensively [1,2,3]. Owing to their reduced weight, small size, high output power, good beam quality, and high electro-optical conversion efficiency, Yb-doped fiber (YDF) lasers and amplifiers have applications in industrial manufacturing [4], laser lidar [5], space debris removal, and free-space optical communication [6]. However, the high laser output is strictly limited by the fact that YDF suffers from two-fold degradation [7,8], commonly referred to as the photodarkening (PD) and radiation darkening (RD) effects [9,10], when operating in amplifying conditions and harsh environments. PD and RD can be induced by pumping and external ionizing radiation, resulting in additional optical loss and a sharp decline in laser output power and slope efficiency.

Researchers generally conclude that the generation of color centers in the fiber is the main reason for the appearance of the darkening effect. Similar loss variations appear after long-term pumping or γ-ray irradiation, indicating that the types of color centers generated in PD and RD are the same [11]. Although the origin and mechanism of color centers are controversial, many studies have confirmed that Yb^2+^ ions and oxygen hole center defects are the main causes of PD and RD in YDF [12].

The main methods to suppress the effects of PD and RD are gas loading and core composition optimization. Many researchers have demonstrated that H_2_-loading is beneficial for improving the radiation resistance of fibers [13,14], but there are some limitations as well. For example, H_2_ molecules can easily escape, the mechanical strength of the coating may decrease, and H_2_ causes additional absorption [15]. Even worse, owing to the reducing environment caused by H_2_, Yb^3+^ ions are easily reduced into Yb^2+^ ions [16]. Core composition optimization is the most fundamental method. Ce co-doping has been used to suppress the formation of aluminum-oxygen hole center (Al-OHC) defects [17,18], and the valence variation of Ce^4+^→Ce^3+^ reduces the generation of Yb^2+^ ions and further improves the radiation resistance. However, this presents the problem that Ce co-doping seriously impacts the spectral properties of Yb^3+^ ions [19]. Jetschke et al. proposed a method to improve the PD resistance by Al/P co-doping [20]. However, it reduces the absorption and emission cross-sections of Yb^3+^ ions, and the radiation resistance of the Yb/Al/P co-doped fiber is relatively weak [21,22]. It has been confirmed that Ge co-doping can reduce the radiation-induced absorption (RIA) caused by Al- and/or P-related defects during irradiation [23,24] and improve the radiation resistance of Er/Al/Ge co-doped glasses and fibers without negatively affecting the spectral properties of Er^3+^ ions [25]. However, the effects of Ge co-doping on the spectral and radiation-resistant properties of Yb-doped silica glasses have not yet been reported.

The absorption band caused by radiation-induced color centers is mainly in the ultraviolet–visible range [12]. Limited by Rayleigh scattering, the absorption of the fiber sample is relatively large in the ultraviolet–visible band; therefore, it is difficult to collect accurate data in the fiber. In addition, continuous-wave electron paramagnetic resonance (CW-EPR) is an effective method for studying paramagnetic color centers. Owing to the small size of the fiber core, it is difficult to obtain powder samples for CW-EPR tests. Therefore, in this work, uniform and large-scale Yb/Al/Ge co-doped silica glasses with different GeO_2_ contents were prepared using the sol–gel method combined with high-temperature powder sintering. By analyzing the absorption, fluorescence spectra, and fluorescence decay curves of the glasses, the effects of Ge co-doping on the Yb^2+^ ion suppression and the Yb^3+^ ion spectral properties were studied. By comparing the changes in luminescence intensity and fluorescence lifetime of Yb^3+^ ions before and after irradiation, the effect of Ge co-doping on the radiation resistance of Yb/Al/Ge co-doped glasses was studied, and the related mechanisms were revealed by RIA and CW-EPR spectra.

## 2. Experimental Details

### 2.1. Sample Preparation

0.15Yb_2_O_3_−1.5Al_2_O_3_−xGeO_2_−(98.35−x)SiO_2_ (in mol%, x = 0, 1, 4, 8, 12) glasses were prepared using the sol–gel method combined with high-temperature powder sintering [26]. The glasses were labeled GAY0, GAY1, GAY4, GAY8, and GAY12 (referred to as GAY samples) based on the theoretical content of GeO_2_. In addition, Al-doped and Ge-doped silica glasses were prepared using the same method for comparative analysis. The actual compositions of the samples were measured using inductively coupled plasma optical emission spectrometry (ICP-OES; radial-view Thermo iCAP 6300, Thermo Fisher Scientific Inc., Waltham, Massachusetts, USA). The theoretical and actual compositions are presented in Table 1. The actual Yb_2_O_3_ content was close to the theoretical values. Even if part of the GeO_2_ volatilized, 50–62% of the theoretical content remained. The actual Al content was higher than the theoretical content because the glasses were polluted by the corundum crucible. The samples were polished into bulk glasses of approximately 10 mm diameter and 2 mm thickness for further testing. The powdered samples, which were utilized for the CW-EPR tests, had masses of approximately 100 mg.

### 2.2. Analyses of Samples

The densities of the samples were measured using an SD-200L electronic densimeter based on the Archimedes drainage method. The refractive indices of the samples were measured using a 2010/M prism coupler (Metricon Corp., Pennington, New Jersey, USA). The absorption spectra were measured using a Perkin-Elmer Lambda 950UV/VIS/NIR spectrophotometer. Fluorescence spectra and fluorescence decay curves were measured using an FLS920 steady/transient state high-resolution fluorescence spectrometer (Edinburgh Instruments Ltd., Kirkton Campus, UK). The fluorescence spectra of Yb^3+^ ions were measured using a xenon lamp under 896 nm excitation. The fluorescence decay curves of Yb^3+^ ions were measured at 1020 nm under excitation by a 980 nm LD. The fluorescence spectra and fluorescence decay curves of Yb^2+^ ions were both measured at 525 nm under the excitation of a 330 nm xenon lamp, and the fluorescence decay curve of the Ge oxygen deficiency center (Ge-ODC) was measured at 395 nm under the excitation of a 330 nm xenon lamp.

To study the effect of GeO_2_ content on the radiation resistance of Yb/Al/Ge co-doped silica glasses, an X-ray instrument (XRad160, Precision X-Ray, Inc., Madison, Wisconsin, USA) was used to irradiate the samples. The total dose and dose rate of irradiation were 3000 Gy and 1440 Gy/h, respectively.

The paramagnetic defects induced by irradiation were recorded using an E-580 Bruker Elexsys X-band EPR spectrometer (Bruker Co., Billerica, Massachusetts, USA). The microwave frequency was approximately 9.38 GHz. All the measurements were conducted at room temperature (300 K).

## 3. Results and Discussion

### 3.1. Effects of GeO_2_ Content on the Spectral Properties of Yb/Al/Ge Co-Doped Silica Glasses

**Figure 1:**
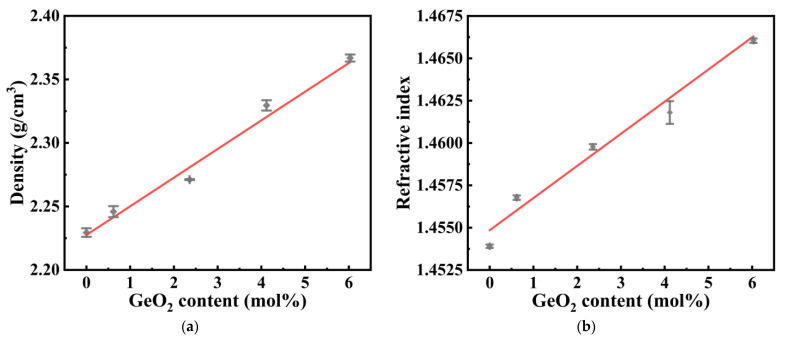
(**a**) Density and (**b**) refractive index of GAY samples.

Figure 1a,b show the density and refractive index of the GAY samples with different GeO_2_ contents. With an increase in GeO_2_ content, the density and refractive index of the samples increased almost linearly. Linear fitting reveals that the slopes of the density and refractive index are approximately 2.26 × 10^−2^ g·cm^−3^·mol^−1^ and 1.85 × 10^−5^ mol^−1^. Although Ge co-doping may cause a higher refractive index of the fiber core, the numerical aperture (NA) of the fiber can still be controlled by creating a graded refractive index layer [27,28].

**Figure 2:**
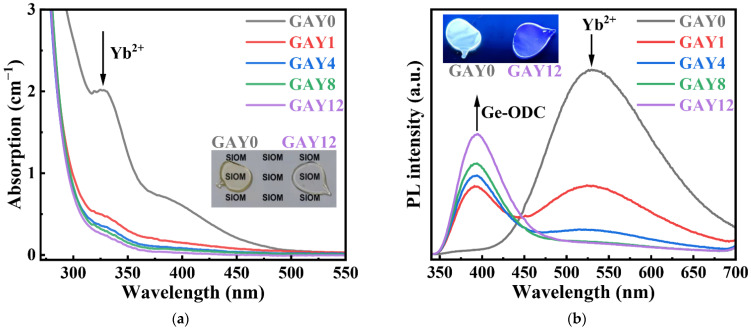
(**a**) Absorption and (**b**) fluorescence of pristine GAY samples; comparison photographs of GAY0 and GAY12 samples (inset in (**a**)) under natural light and (inset in (**b**)) under ultraviolet light.

Figure 2a shows the absorption spectra of the pristine GAY samples with different GeO_2_ contents in the range of 270–550 nm. The Ge-free (GAY0) sample has an absorption band at 330–450 nm, which is primarily attributed to the 4f–5d transition of Yb^2+^ ions [29,30]. With an increase in the GeO_2_ content, the absorption intensity of the Yb^2+^ ions is significantly reduced. Even in the GAY1 sample (GeO_2_ content of 0.62 mol%), the absorption intensity of Yb^2+^ ions is very weak. This shows that a low doping concentration of Ge can effectively suppress the formation of Yb^2+^ ions. As shown in the inset of Figure 2a, owing to the higher absorption intensity of Yb^2+^ ions, the GAY0 sample was yellowish. In contrast, the GAY12 sample was colorless and transparent.

Figure 2b shows the fluorescence spectra of the pristine GAY samples in the range of 340–700 nm excited by a 330 nm xenon lamp. The fluorescence spectrum of the GAY0 sample has a very broad emission band at 525 nm, which was caused by Yb^2+^ ion emission [29], and it is primarily attributed to the 4f–5d transition of Yb^2+^ ions [30]. With an increase in the GeO_2_ content, the fluorescence intensity of Yb^2+^ ions decreased, and a new fluorescence peak appeared and increased simultaneously at 395 nm, which can be attributed to the Ge-ODC defect emission [31]. The absorption of Ge-ODC mainly originates from the S0→S1 transition, and the emission of Ge-ODC primarily originates from the transition of T1→S0. The energy level diagram of Ge-ODC can be found in Ref. [32]. As shown in the inset of Figure 2b, the GAY0 sample emits yellowish light under ultraviolet light, which is mainly attributed to the emission of Yb^2+^ ions. Meanwhile, mainly attributed to the emission of Ge-ODC defects, the GAY12 sample emits bluish light. These results further confirm that Ge co-doping can effectively suppress the generation of Yb^2+^ ions.

**Figure 3:**
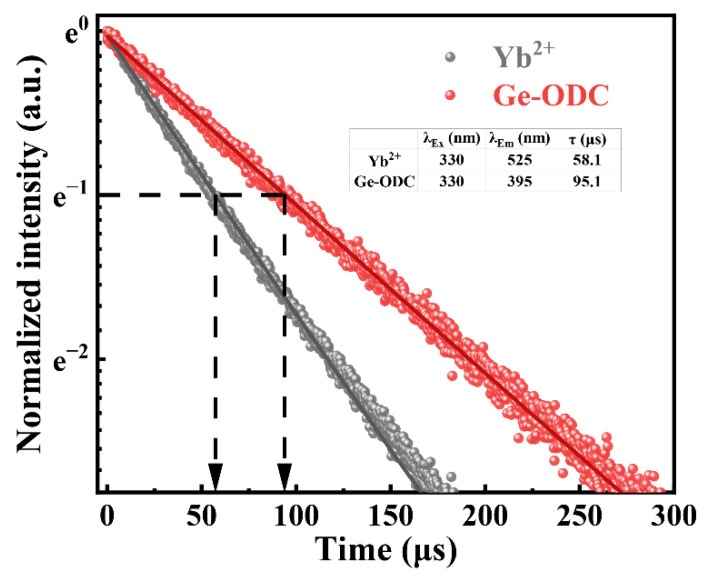
Fluorescence decay curves of Yb^2+^ ions and Ge-ODC.

Figure 3 shows the fluorescence decay curves of Yb^2+^ ions and Ge-ODC. The lifetime of Yb^2+^ ions measured at 525 nm under 330 nm excitation with the GAY0 sample is 58.1 μs [33]. The lifetime of Ge-ODC measured at 395 nm under 330 nm excitation with a high GeO_2_ content (GAY12) sample is 95.1 μs. The results confirm that the fluorescence peaks at 525 nm and 395 nm (see Figure 2b) originate from two different fluorescence centers of Yb^2+^ and Ge-ODC, respectively.

**Figure 4:**
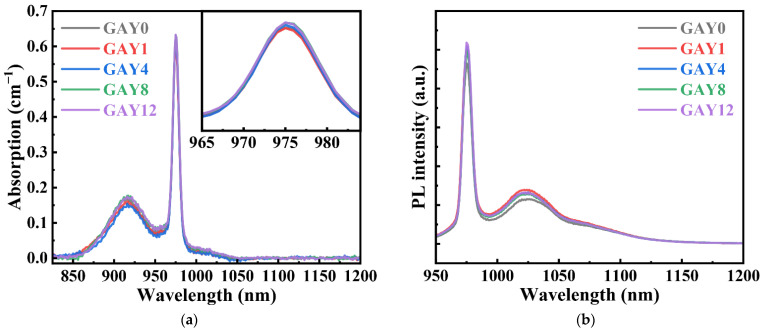
(**a**) Absorption spectra, (inset: enlargement in the range of 965–984 nm), (**b**) Fluorescence spectra of pristine GAY samples.

Figure 4a,b show the absorption and fluorescence spectra of Yb^3+^ ions in the GAY samples. The absorption and emission of Yb^3+^ primarily originates from 4f–5d transition [30]. The absorption curves of the samples with different GeO_2_ contents roughly overlap, and the absorption intensities of the samples at 976 nm are almost the same. In addition, the fluorescence spectra of the samples under 896 nm excitation roughly coincide. The fluorescence intensity of the Ge co-doped samples is slightly higher than that of GAY0. This may be due to the suppression of Ge co-doping with Yb^2+^ ions. This proves that Ge co-doping has no negative impact on the absorption and fluorescence spectra of Yb^3+^ ions.

**Figure 5:**
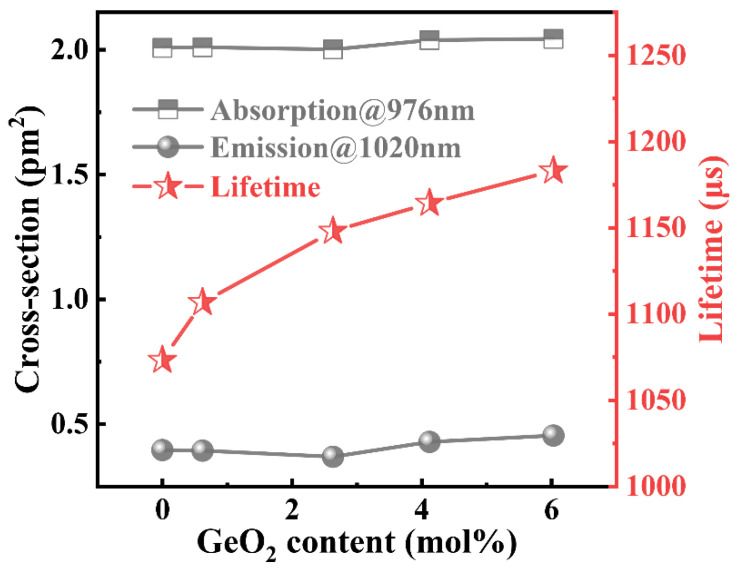
Absorption, emission cross-section, and fluorescence lifetime of Yb^3+^ ions in pristine GAY samples.

Figure 5 shows the absorption cross-section at 976 nm, emission cross-section, and fluorescence lifetime at 1020 nm of Yb^3+^ ions. The absorption and emission cross-sections were calculated using the Lambert–Beer law and the Fuchtbauer–Lademnurg (F–L) formula [34]. With an increase in the GeO_2_ content, the absorption cross-section and emission cross-section of Yb^3+^ ions hardly changed, but the fluorescence lifetime of Yb^3+^ ions increased gradually.

The formation mechanism of Yb^2+^ ions in Yb/Al co-doped silica glasses during vacuum sintering may be related to the disappearance of Al-related oxygen deficiency centers (Al-ODC). The corresponding chemical reactions are as follows:

(1) $${\mathrm{Yb}}^{3+}+\begin{array}{c} \equiv {\mathrm{Al}}^{••}\mathrm{Si}\equiv \\ \left(\mathrm{Al}-\mathrm{ODC}\right) \end{array}\overset{\Delta /\mathrm{vacuum}}{\to }{\mathrm{Yb}}^{2+}+\begin{array}{c} \equiv {\mathrm{Al}}^{•}{}^{\circ}\mathrm{Si}\equiv \\ \left(\mathrm{Al}-E^{\prime }\right) \end{array}$$

where, “^•^” and “^°^” represent an electron and a hole, respectively. This speculation is supported by the results of Kirchhof et al. [30]. Al-, P-, and Ge-related oxygen defect centers (ODC) are easily formed during sintering in a vacuum or inert atmosphere (e.g., He). However, Kirchhof et al. found that in 6P_2_O_5_-94SiO_2_ and 7GeO_2_-93SiO_2_ (in mol%) glasses sintered in an inert atmosphere, the introduction of Yb^3+^ ions did not significantly change the fluorescence intensity of Ge-ODC and P-ODC, and no Yb^2+^ ion fluorescence was detected. In contrast, in 4Al_2_O_3_-0.5P_2_O_5_-95.5SiO_2_ (in mol%) glasses sintered in an inert atmosphere, the introduction of Yb^3+^ ions significantly reduced the fluorescence intensity of Al-ODC, and meanwhile, the fluorescence of Yb^2+^ ions was detected. Herein, with an increase in the GeO_2_ content, Ge-ODC became the main ODC defect at the expense of Al-ODC. Therefore, Yb^2+^ ions were gradually inhibited.

### 3.2. Effects of GeO_2_ Content on the Radiation Resistance of Yb/Al/Ge Co-Doped Silica Glasses

**Figure 6:**
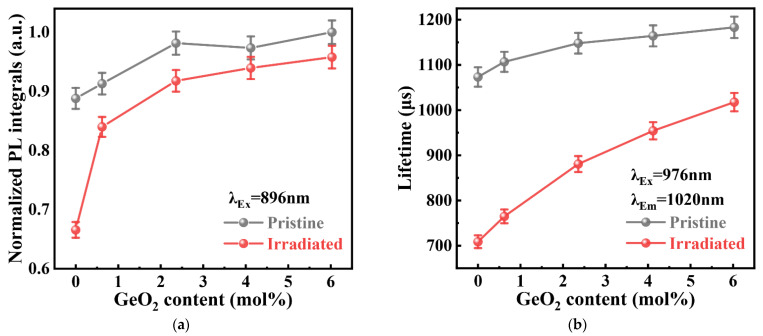
(**a**) Fluorescence integral intensity and (**b**) fluorescence lifetime of GAY samples before and after irradiation.

Figure 6a,b show the fluorescence integral intensity in the range of 950–1200 nm and fluorescence lifetime at 1020 nm of the GAY samples before and after irradiation. Before irradiation, with an increase in the GeO_2_ content, both the fluorescence integral intensity and fluorescence lifetime of Yb^3+^ ions increased slightly. The increase in the fluorescence integral intensity may be related to the oxidation of Yb^2+^ ions to Yb^3+^ ions. The increase in the fluorescence lifetime may be related to the low phonon energy caused by the coordination of Ge to Yb^3+^ ions. After irradiation, the fluorescence integral intensity and fluorescence lifetime of all samples decreased. The fluorescence integral intensity and fluorescence lifetime of the GAY0 sample showed the largest decrease. Meanwhile, with increasing GeO_2_ content, the difference decreased. This result shows that Ge co-doping can effectively improve the radiation resistance of Yb/Al co-doped silica glasses.

Previous studies have shown that the darkening effect of YDF is related to the valence variations of Yb^2+/3+^ ions and the formation of dopant-related point defects [16,21]. Compared with Yb^2+^ ions, point defects have a larger absorption cross-section and wider absorption band, and they are closer to the absorption and emission wavelengths of Yb^3+^ ions; therefore, they significantly impact the spectral properties of Yb^3+^ ions.

**Figure 7:**
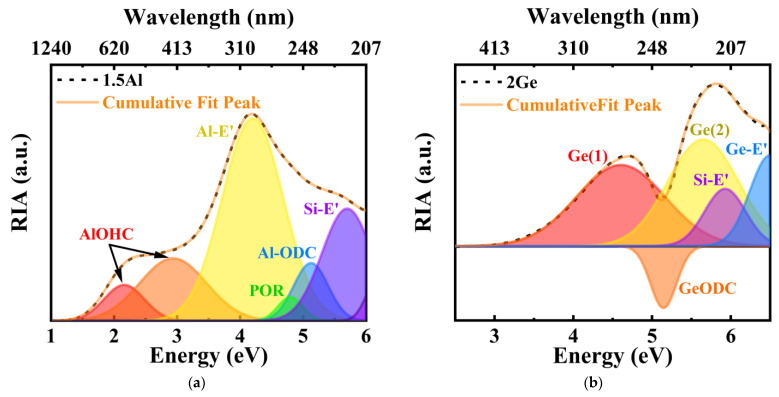

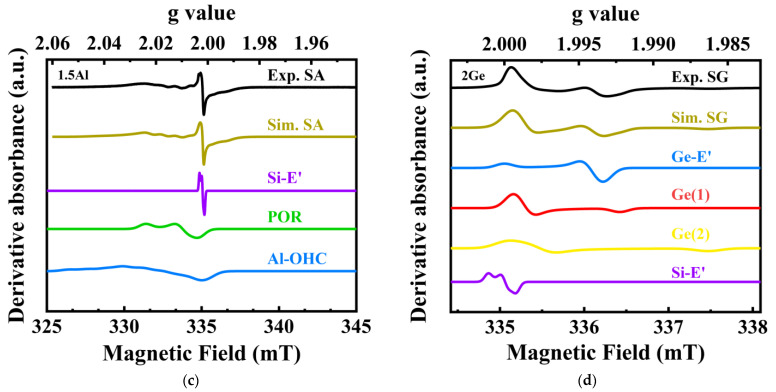
Gaussian decomposition of RIA of (**a**) Al-doped and (**b**) Ge-doped glasses; CW-EPR spectra of irradiated (**c**) Al-doped and (**d**) Ge-doped glasses.

The Al-doped and Ge-doped silica glasses without Yb^3+^ ions were prepared to eliminate the contribution of Yb^2+^ ion absorption to the RIA (see Figure 7a,b). The cumulative fitted peaks were consistent with the observed RIA. The RIA spectrum of the Al-doped sample was decomposed into six Gaussian components with peaks at 2.2, 2.9, 4.2, 4.8, 5.1, and 5.8 eV. These bands can be attributed to the Al-OHC defect (≡Al-O°, 2.2 and 2.9 eV) [35], aluminum dangling bond (Al-E’) defect (4.2 eV) [35], peroxy radical (POR) defect (4.8 eV) [35], Al-ODC defect (5.1 eV), and silicon dangling bond (Si-E’) defect (5.8 eV) [36]. The RIA spectrum of the Ge-doped sample was decomposed into five Gaussian components with peaks at 4.6, 5.1, 5.6, 5.8, and 6.4 eV. These bands can be attributed to the Ge(1) defect (4.6 eV) [37], Ge-ODC (5.1 eV) [38], Ge(2) defect (5.6 eV) [39], Si-E’ defect (5.8 eV) [36], and germanium dangling bond (Ge-E’) defect (6.4 eV) [40]. The RIA intensity of the Ge-ODC defects was negative, which means that the content of Ge-ODC decreased after irradiation. The RIA intensities of the other defect centers were all positive, which means that the contents of these defects increased after irradiation.

Figure 7c,d show the CW-EPR spectra of the Al-doped and Ge-doped silica glasses after irradiation. The CW-EPR spectrum of the Al-doped sample was decomposed into three parts, corresponding to the POR, Si-E’, and Al-OHC defect centers. Theoretically, owing to the hyperfine coupling interaction between the holes and the magnetic core ^27^Al (I = 5/2, NA~100%), six hyperfine lines can be observed at each g component (g_1_, g_2_, g_3_, 2 I + 1 = 6). However, since the hyperfine lines of the g components are superimposed on each other, this phenomenon is difficult to observe experimentally. The CW-EPR spectrum of the Ge-doped samples was decomposed into four parts, corresponding to the Ge-E’, Ge(1), Ge(2), and Si-E’ defect centers. Since the natural abundance of the magnetic core ^73^Ge is only 7.76%, no hyperfine lines for the Ge-E’, Ge(1), or Ge(2) defects are observed in this EPR spectrum.

Al-ODC and Ge-ODC are diamagnetic centers (no CW-EPR signal) and cannot be detected in the CW-EPR test. Although the Al-E’ defect is a paramagnetic center, since its spin–lattice relaxation time is relatively long, it is usually detectable only in the scattering (non-absorption) mode and very low microwave power [36]. Therefore, the CW-EPR signal of the Al-E’ defect cannot be observed in Figure 7c.

**Figure 8:**
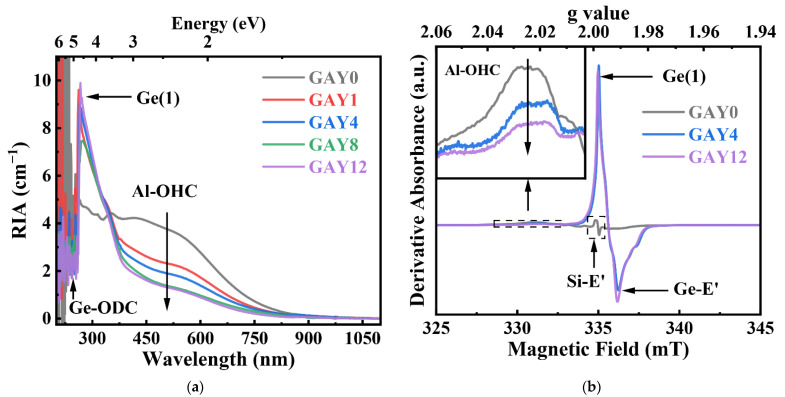
(**a**) RIA and (**b**) CW-EPR spectra of irradiated GAY samples.

Figure 8a,b show the RIA and CW-EPR spectra of the GAY samples. In contrast to the fitting results in Figure 7, the absorption of the RIA at 540 nm in Figure 8a is mainly attributed to Al-OHC, and the RIA in the ultraviolet band is mainly attributed to Ge-related defects. In Figure 8b, the signal of CW-EPR at 331 mT is also mainly attributed to Al-OHC defects, and the CW-EPR signal in the central region (334–337 mT) is mainly attributed to Ge-related defects. Figure 8a,b show that with an increase in the GeO_2_ content, the RIA and CW-EPR intensity of the Al-OHC decreased significantly, and the RIA and CW-EPR intensity of the Ge-related defects gradually increased. Compared with Ge-related defects, the absorption band of Al-OHC defects is closer to the infrared band, and the extension of its absorption band can even cover 1 μm. Therefore, compared to Al-OHC, Ge-related defects have a slighter impact on the spectral properties of Yb^3+^ ions.

Previous studies have shown that in Yb/Al co-doped silica glasses, Yb^3+^ ions are coordinated in [AlO_4/2_]^−^ tetrahedra, and the following reactions occur during the irradiation process [21,25]:

(2) $${\mathrm{Yb}}^{3+}-O-\mathrm{Al}\equiv +e^{-}+h^{+}\overset{h\nu }{\to }{\mathrm{Yb}}^{2+}+\begin{array}{c} {}^{\circ}O-\mathrm{Al}\equiv \\ \left(\mathrm{Al}-\mathrm{OHC}\right) \end{array}$$

In Yb/Al/Ge co-doped silica glasses, the content of Ge-ODC increases with increasing GeO_2_ content. There are two types of Ge-ODC color centers (Ge-ODC(I) and Ge-ODC(II)), which can be converted into each other during the irradiation process. The presence of Ge-ODC was confirmed by the fluorescence spectra (see Figure 2b). Since Ge-ODC(I) has a stronger ability to capture holes than the [AlO_4/2_]^−^ group [41], Ge co-doping enables Ge-ODC(I) to capture more holes, thereby increasing the radiation-induced Ge-E’ content, as follows:

(3) $$\begin{array}{c} 2\left[={\mathrm{Ge}}^{•}\right]\\ \left(\mathrm{Ge}-\mathrm{ODC}\left(\mathrm{II}\right)\right) \end{array}\overset{h\nu }{↔}\begin{array}{c} \equiv \mathrm{Ge}-\mathrm{Ge}\equiv \\ \left(\mathrm{Ge}-\mathrm{ODC}\left(I\right)\right) \end{array}+h^{+}\overset{h\nu }{\to }\begin{array}{c} \equiv {\mathrm{Ge}}^{•}{}^{\circ}\mathrm{Ge}\equiv \\ \left(\mathrm{Ge}-E^{\prime }\right) \end{array}$$

Meanwhile, the number of holes captured by the [AlO_4/2_]^-^ group is reduced, thereby reducing the Al-OHC content. Figure 8a shows a cavity that appears in the RIA curve at 248 nm, which indicates that the Ge-ODC content decreases after irradiation. With an increase in the GeO_2_ content, the intensity of the RIA curve weakens at 540 nm, which means that the Al-OHC defect content decreases. In addition, as the number of [GeO_4/2_]^0^ groups increases with increasing GeO_2_ content, additional electrons are captured by the [GeO_4/2_]^0^ groups to form Ge(1) and Ge(2) color centers, and the formula is as follows:

(4) $$\begin{array}{c} \equiv \mathrm{Ge}-O-M\equiv \\ \left({\left[{\mathrm{GeO}}_{4/2}\right]}^{0}\right) \end{array}+e^{-}\overset{h\nu }{\to }\begin{array}{c} \equiv {\mathrm{Ge}}^{•}-O-M\equiv \\ \left(\mathrm{Ge}\left(1\right)\;\mathrm{or}\;\mathrm{Ge}\left(2\right)\right) \end{array}\left(M=\mathrm{Si},\;\mathrm{Al}\;\mathrm{or}\;\mathrm{Ge}\right)$$

Figure 8a shows that the intensity of the RIA curve increases significantly with an increase in the GeO_2_ content at 269 nm, which indicates an increase in the Ge(1) defect content. Figure 8b shows that the CW-EPR intensity of Ge(1) and Ge-E’ defects increases with increasing GeO_2_ content. This process suppresses the formation of other trapped-electron centers (Yb^2+^/Si-E’/Al-E’).

## 4. Conclusions

Herein, the effects of Ge co-doping on the valence state of Yb^2+/3+^ ions, spectral properties of Yb^3+^ ions, and radiation resistance of Yb/Al/Ge co-doped silica glasses were studied systematically. For the pristine GAY samples, with an increase in the GeO_2_ content, the generation of Yb^2+^ ions was considerably suppressed, and the spectral properties of Yb^3+^ ions were improved slightly. After X-ray irradiation, the RIA and CW-EPR spectra confirmed that the Al-OHC defects were effectively inhibited by Ge co-doping. In addition, the fluorescence integral intensity and fluorescence lifetime results also confirmed that the radiation resistance of the samples was improved with increasing GeO_2_ content.

The generation and suppression mechanism of Yb^2+^ ions in the pristine Yb/Al/Ge co-doped silica glasses and color centers in irradiated samples are discussed. For the pristine samples, Al-ODC trapped holes to form Al-E’ defects during high-temperature sintering. With increasing GeO_2_ content, Ge-ODC became the main ODC defect at the expense of Al-ODC. Thus, the relatively stable Ge-ODC inhibited the process by which Yb^3+^ ions trap electrons to form Yb^2+^ ions. When the Yb/Al/Ge co-doped silica glasses were irradiated, the [AlO_4/2_]^−^ groups trapped holes to form Al-OHC, and Yb^3+^ ions trapped electrons to form Yb^2+^ ions. Since Ge-ODC has a stronger ability to capture holes than [AlO_4/2_]^−^ groups, the formation of trapped-hole centers (Al-OHC) was inhibited. With an increase in the GeO_2_ content, the increasing [GeO_4/2_]^0^ groups formed Ge(1) and Ge(2) color centers by trapping electrons. This process inhibited the formation of all other types of trapped-electron centers (Yb^2+^/Si-E’/Al-E’). This work suggests that Ge co-doping can be effective for suppressing the generation of Yb^2+^ ions and improving the radiation resistance of Yb-doped silica glasses.

## Figures and Tables

**Table 1:** Theoretical and actual compositions of the GAY, Al-doped, and Ge-doped samples.

Samples	Theoretical Composition (in mol%)			Actual Composition (in mol%)		
	GeO_2_	Yb_2_O_3_	Al_2_O_3_	GeO_2_	Yb_2_O_3_	Al_2_O_3_
1.5Al	0	0	1.5	0	0	2.11
2Ge	2	0	0	1.17	0	0
GAY0	0	0.15	1.5	0	0.16	2.01
GAY1	1	0.15	1.5	0.62	0.16	2.45
GAY4	4	0.15	1.5	2.36	0.16	2.10
GAY8	8	0.15	1.5	4.12	0.15	2.21
GAY12	12	0.15	1.5	6.03	0.16	2.33

## Data Availability

The data that support the findings of this study are contained within the article.
